# XEN Gel Stent Early Failure-dye-enhanced Ab-externo Revision

**DOI:** 10.5005/jp-journals-10028-1258

**Published:** 2018

**Authors:** Nuno P Ferreira, Joana M Pinto, Filipa Teixeira, Luís A Pinto

**Affiliations:** 1 Department of Ophthalmology, Hospital Santa Maria, Lisboa Norte, Lisbon, Portugal; 2 Visual Sciences Study Center, Faculdade de Medicina da Universidade de Lisboa, Lisbon, Portugal; 3 Hospital Divino Espirito Santo, Ponta Delgada, Açores, Lisboa, Portugal

**Keywords:** Complications, Glaucoma surgery, Minimally invasive glaucoma surgery, XEN stent

## Abstract

The XEN gel stent is an ab-interno minimally invasive glaucoma surgery device that reduces intraocular pressure by creating a subconjunctival drainage pathway. XEN intents to provide a safer and less invasive mean of lowering intraocular pressure. As with any new device, there is still some lack of experience and knowledge concerning efficacy, technique, and complications. We report a novel surgical approach for early bleb failure after XEN implantation.

**How to cite this article:**

Ferreira NP, Pinto JM, Teixeira F, Pinto LA. XEN Gel Stent Early Failure-dye-enhanced Ab-externo Revision. J Curr Glaucoma Pract 2018;12(3):139-141.

## SURGICAL TECHNIQUE

 

## INTRODUCTION

Minimally invasive glaucoma surgery (MIGS) aims to provide a safer and less invasive procedure to reduce intraocular pressure (IOP) comparing to traditional surgery.^1^ To date, available MIGS procedures offer more modest results regarding IOP-lowering efficiency than traditional glaucoma surgery, but with the benefit of a safer risk profile, being currently targeted for patients with mild-to-moderate glaucoma.^1^ XEN gel stent (Allergan Inc. Dublin, Ireland) is an *ab-interno* MIGS device provides a subconjunctival drainage pathway.^2^ As with any new device, there is still some lack of experience and knowledge concerning efficacy, technique, and complications.^1^ We report a novel surgical approach for early bleb failure after XEN implantation.

## CASE DESCRIPTION

### History of the Disease

A 67 years-old Caucasian male, bilaterally pseudophakic, followed in the glaucoma clinic of the Hospital de Santa Maria due to bilateral open-angle glaucoma secondary to pseudoexfoliation, presented with visual field deterioration in his right eye (OD). His IOP was above target (24mmHg) despite maximum medical treatment (bimatoprost, timolol, and dorzolamide) and due to patient concerns regarding trabeculectomy, a less invasive approach was discussed, and implant of ab-internal XEN collagen device was proposed under peribulbar anesthesia. The procedure was uneventful: mitomycin C (MMC, 1 mL at 0.02%) was injected subconjunctivally on the superonasal sector with subsequent XEN 45 device being was implanted ab-interno. On the early postoperative period, there were no complications, and target IOP was achieved under no topical treatment. After three months follow-up, the patient presented with high IOP (26 mm Hg) and with a thickened, non-diffuse bleb, so the patient was started on timolol bid. One week later, having no clinical improvement, it was decided to proceed with the surgical revision under topical anesthesia.

### Needling Surgical Technique

**Figs 1A and B:**
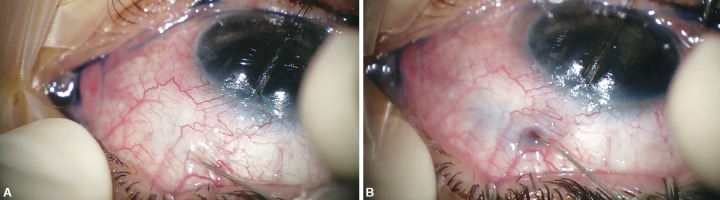
(A) After performing a paracentesis trypan blue is injected in the anterior chamber; (B) The trypan blue is useful to assess if any residual subconjunctival drainage is present. If the procedure is being effective a diffuse bluish diffusion is seen through the subconjunctival space

Needling of the bleb assisted with vital dye was performed. The employed technique and postoperative care are described (Video 1):

- Injection in the anterior chamber of trypan blue 0.1%, 0.1 mL (Vision Blue, DORC international, BV Zuidland, Netherlands). The trypan blue is meant to highlight any subconjunctival drainage (Fig. 1A).
- A 30-gauge bent needle is then inserted in the subconjunctival space, posterior to the distal end of the XEN, about 7 mm distal from the limbus.
- The needle is slowly advanced under the conjunctiva until it encounters the space near the XEN distal outflow. Any resistance was managed with careful side-to-side as to-and-fro movements, to release any fibrosis. At this time, if the procedure is being effective, a diffuse bluish diffusion is seen through the subconjunctival space (Fig. 1B). Special care must be taken not to buttonhole the conjunctiva during the procedure.
- A volume of MMC 0.02 mL (0.2 mg/mL) is then injected posteriorly near the intended site of revision.
- Wash out of the anterior chamber with a balanced salt solution.
- Close the paracentesis incisions by stromal hydration.
- Postoperatively the patient is started on topical prednisolone and antibiotic four times daily for 4 weeks.

## RESULTS

One week after surgery the patient showed controlled IOP (10 mm Hg) and an open diffuse filtering bleb. Two months after surgery the intraocular pressure was under control (13 mm Hg) with no IOP-lowering medications.

## DISCUSSION

The XEN is a collagen stent introduced via ab-interno, through the trabecular meshwork, with the goal of reducing IOP by creating a subconjunctival drainage pathway.^1,3^ The XEN 45 implant has around 45 µm inner lumen, and the combination of length and internal lumen diameter provides roughly 6–8 mm Hg of resistance to flow, which substantially eliminates hypotony, as calculated by the Hagen-Poiseuille equation and verified by laboratory flow testing.^2,4^ The XEN is implanted via a clear corneal incision without conjunctival dissection.^1,2^ Currently, this procedure is being used in patients with mild-to-moderate glaucoma, and often it is combined with cataract surgery. The first results reported by Pérez-Torregrosa et al. were promising.^5^ They performed combined phacoemulsification with XEN 45 implant surgery, on patients with at least two antiglaucoma medications, and an IOP reduction of 29.34% was seen after 1-year follow-up. The number of antiglaucoma medications reduced 94.57%. In all cases cataract surgery was unremarkable. Regarding complications, they only reported one eye with bleb encapsulation which was controlled with topical lowering IOP medication.^5^

Sheybani and coworkers also reported promising results of combined cataract surgery with the XEN 45 and XEN 140.^6^ They obtained an IOP reduction by a mean of 7.0 mm Hg at 12 months’ follow-up and achieved a reduction in the use of medicaments of 50%. Twelve patients (32%) required a needling procedure with anti-metabolic agents. Hypotony below 5mm Hg was seen in 13 patients not exceeding the first month after surgery (9 of which had the XEN 140). In 2016, Sheybani et al. published the results of XEN140 implant without intraoperative adjunctive MMC. At 12 months, 89% of patients achieved an IOP ≤ 18 mmHg, and 40% of patients were medication-free.^7^ However, the needling rate was higher compared with the results with adjunctive MMC, with 21 patients requiring needling (47%).

Bashford and Lavin-Dapena compared cataract surgery and XEN implantation with standalone XEN procedure. From those that achieved a 12-month follow-up, 79.5% and 86.4% had a 20% reduction of IOP, respectively.^8^ Eight patients needed a second surgical procedure related to the XEN implant, and four patients had an implant blockage.^8^

The available published results related to intraoperative complications are variable. From our experience, needling of a XEN bleb is a relatively common procedure and every ophthalmologist should be able of handling it. The use of a vital dye can be a useful tool for helping the surgeon to see if the manipulation of the subconjunctival is effective.

There are two ongoing clinical trials with the aim of studying XEN implantation for the treatment of moderate primary open-angle glaucoma patients when medications are inaccurate (NCT02006693), while another one is investigating the outcomes of the same device in patients with refractory glaucoma (NCT02036541).^6^ After the results of these studies, we will possibly have more clear indications for XEN implantation. Until then, experience and selective patient recruitment are key to achieve the best results.

Trypan blue has been an essential adjunctive surgical agent due to its safety, ease of use, and mainly by making surgical procedures safer and easy to perform.^9,11^ Its action is based on the capability of highlighting biological tissues. Besides using trypan blue in cataract surgery and corneal transplantation procedures, it has been used as an adjunctive dye in glaucoma procedures.^12,13^ Rodriguez et al. did a review of vital dies in ocular surgery. They stated that goals when using a dye in glaucoma surgery are an evaluation of the patency of glaucoma filtering blebs and safety by coloring the anti-metabolic agents.^14^

To our best knowledge, this is the first case reporting the surgical technique of a dye-enhanced XEN gel stent bleb needling.

## CONCLUSION

As with any new surgical procedure, it is essential to know how to manage the complications brought by these new devices. Vital dye assisted needling of the XEN bleb seems to enhance intra-operative assessment of the success of the revision procedure.

## CLINICAL SIGNIFICANCE

This is the first case reporting the surgical technique of a vital dye assisted needling of a XEN implant early failure.
